# Editorial: Improving yield and quality of cereal crops: exploring and utilizing genes for green and efficient traits

**DOI:** 10.3389/fpls.2025.1617702

**Published:** 2025-06-10

**Authors:** Hao Zhou, Yuqing He, Ming Luo

**Affiliations:** ^1^ State Key Laboratory of Cop Gene Exploration and Utilization in Southwest China, Rice Research Institute, Sichuan Agricultural University, Chengdu, China; ^2^ National Key Laboratory of Crop Genetic Improvement and National Centre of Plant Gene Research (Wuhan), Huazhong Agricultural University, Wuhan, China; ^3^ Agriculture and Food, Commonwealth Scientific and Industrial Research Organisation (CSIRO), Canberra, ACT, Australia

**Keywords:** cereal crop, green and efficient trait, gene discovery, molecular network, breeding utilization

In the face of escalating global challenges—population growth, climate change, and limited arable land—enhancing the yield and quality of cereal crops has become a cornerstone of sustainable agriculture. This Research Topic delves into the genetic and molecular mechanisms underpinning stress resilience, nutritional improvement, and breeding innovation in staple crops such as rice (*Oryza sativa*), wheat (*Triticum aestivum*), and maize (*Zea mays*). By integrating cutting-edge genomic technologies, transcriptomic analyses, and artificial intelligence-driven methodologies, the studies presented here illuminate pathways toward precision breeding for green and efficient traits (**Figure 1**). Below, we contextualize these contributions within broader scientific and agricultural landscapes.

**Figure 1:**
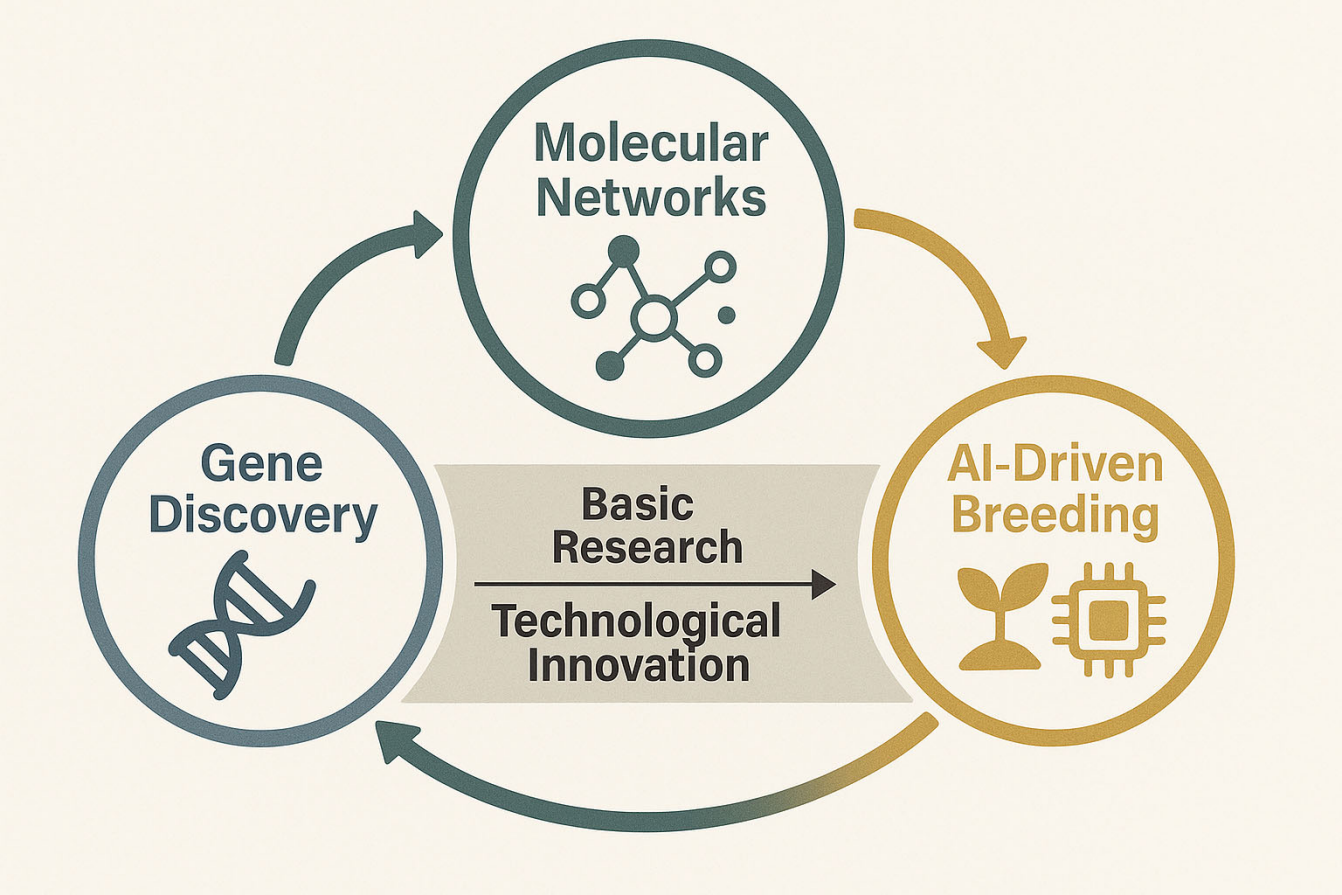
A conceptual diagram of this topic.

## Unlocking stress resilience: from genes to fields

Environmental stressors, including soil compaction, pathogen attacks, and herbicide pressure, persistently threaten cereal production. The featured studies leverage multi-omics approaches to decode plant responses to these challenges. For instance, genome-wide association studies (GWAS) in rice (Klinsawang et al.) identified ‘F-box’ genes (*Os10g0124700*, *Os10g0126600*, and *Os10g0128200*) that modulate ethylene signaling and root architecture under compacted soil conditions. These findings offer actionable targets for breeding rice varieties with enhanced root plasticity, a trait critical for nutrient acquisition in degraded soils. Similarly, transcriptomic profiling of Fusarium head blight (FHB)-resistant wheat landrace *Wuyangmai* revealed miRNA-mediated regulation of glutathione metabolism and phenylpropanoid biosynthesis, pathways pivotal for oxidative stress mitigation and pathogen defense (Wu et al.). Such insights not only deepen our understanding of plant immunity but also highlight miRNAs as novel tools for engineering disease-resistant crops.

Equally compelling is the GWAS-based dissection of herbicide resistance in rice, which uncovered geographically localized alleles (e.g., *RGlu6* and *RGly8*) in European *japonica* varieties (Xu et al.). These results underscore the importance of preserving genetic diversity in germplasm collections to address region-specific agricultural challenges. Collectively, these studies exemplify how genetic diversity and molecular networks can be harnessed to fortify crops against biotic and abiotic threats.

## Quality traits: balancing nutrition and palatability

Crop quality is a multidimensional trait shaped by complex metabolic and regulatory networks. The functional characterization of *OsG6PGH1* in rice exemplifies this complexity (Peng et al.). By interacting with *OsAAP6*, this gene enhances protein body formation and grain protein content while reducing chalkiness, thereby improving both nutritional and sensory properties. Notably, *OsG6PGH1* also contributes to salt stress tolerance, illustrating the pleiotropic roles of genes at the intersection of quality and resilience. Another breakthrough comes from the triple mutant *sbe2b/sbe1/OE-Wxa*, which elevates resistant starch (RS) content to 4.63%—a fivefold increase over wild-type levels—without compromising yield (Chen et al.). This achievement demonstrates the power of stacking mutations in starch biosynthesis genes to achieve synergistic improvements in health-promoting traits and agronomic performance.

## Innovations in breeding technology: from data to decisions

Translating genetic discoveries into breeding outcomes requires robust technological frameworks. The development of *TAL-SRX*, a machine learning model for KASP primer evaluation, exemplifies this transition (Chen et al.). By integrating deep learning (Transformer, LSTM) and traditional algorithms, this tool achieves 92.83% accuracy in genotyping, significantly accelerating marker-assisted selection. Similarly, genomic prediction models (e.g., rrBLUP, LightGBM) applied to maize hybrids in Southwest China identified Reid+ × Suwan+ as the optimal heterotic pattern, aligning computational predictions with empirical breeding outcomes (Xiang et al.). These advances highlight the transformative potential of AI in democratizing precision breeding.

Complementing these tools is the novel strategy for constructing “trait-customized core collections” in winter wheat (Berkner et al.). By balancing phenotypic extremes and genetic diversity, this approach maximizes statistical power in association studies, enabling efficient mining of rare alleles for traits like yellow rust resistance. Such methodologies are indispensable for leveraging genebank resources in the era of big data.

## The trade-off conundrum: immunity vs. agronomic performance

A recurring theme in crop improvement is the trade-off between stress resistance and yield or quality. The rice *spl-A*mutant, harboring a *LRD6-6* mutation, epitomizes this dilemma (Yin et al.). While the mutation confers broad-spectrum disease resistance, it disrupts vesicle trafficking via ESCRT-III interactions, leading to compromised grain quality. This study serves as a cautionary tale: single-gene interventions may incur unintended costs, necessitating strategies such as tissue-specific expression or epistatic modulation to decouple desirable traits from fitness penalties.

## Future directions: integrating disciplines for sustainable agriculture

The studies in this Research Topic collectively underscore the importance of interdisciplinary integration—genomics, bioinformatics, and agronomy—to address food security challenges. Future efforts should prioritize:

1. Precision editing: Combining CRISPR-based allele replacement with multi-omics data to fine-tune trait expression.
2. Dynamic network analysis: Elucidating crosstalk between stress-response, metabolic, and developmental pathways.
3. Climate-resilient prediction models: Leveraging AI to predict genotype × environment × management (G×E×M) interactions.

By bridging fundamental research and translational breeding, we can accelerate the development of “smart” crop varieties that meet the dual demands of productivity and sustainability.

